# Genome-Wide Characterisation of the Ashanti Dwarf Pig Within a Global Context: Insights into Diversity, Inbreeding, and Adaptive Signatures

**DOI:** 10.3390/life16050745

**Published:** 2026-04-30

**Authors:** Sethlina Naa Dodua Aryee, Dennis Owusu-Adjei, Richard Osei-Amponsah, Benjamin Matthew Skinner, Julien Bauer, Benjamin Ahunu, Anton Enright, Carole Anne Sargent

**Affiliations:** 1 Institute for Social and Economic Research, School of Life Sciences, University of Essex, Colchester P.O. Box 1531, UK; b.skinner@essex.ac.uk; 2 Animal Directorate, Ministry of Food and Agriculture, Accra P.O. Box M 37, Ghana; dennis30009@yahoo.com; 3 Department of Animal Science, University of Ghana, Accra P.O. Box LG 25, Ghana; rosei-amponsah@ug.edu.gh (R.O.-A.); ahunubk@ug.edu.gh (B.A.); 4 Department of Pathology, University of Cambridge, Cambridge P.O. Box 338, UK; jb393@cam.ac.uk (J.B.); aje39@cam.ac.uk (A.E.); cas1001@cam.ac.uk (C.A.S.)

**Keywords:** indigenous pigs, genetic resources, linkage disequilibrium, runs of homozygosity, admixture, selection signatures, conservation

## Abstract

Indigenous pig breeds represent valuable reservoirs of genetic diversity but face increasing risks of genetic erosion due to uncontrolled crossbreeding with commercial lines. The Ashanti Dwarf Pig (ADP) of Ghana is an important local genetic resource well-adapted to tropical environments but poorly characterised at the genomic level. Using high-density SNP data from the ADPs and publicly available datasets from other African, European, and Asian pig populations, we examined genetic diversity, population structure, inbreeding, and selection signatures. After quality control, 59,124 SNPs across 875 individuals were retained. ADPs exhibited high polymorphism (~99%) and moderate heterozygosity but also elevated inbreeding (FIS = 0.15; FROH = 0.40), indicating recent inbreeding under free-range management. Population structure revealed that ADPs cluster closely with other African pigs and European breeds more than Chinese breeds. ADMIXTURE analysis, however, indicated recent introgression from both European and Chinese lines. Selection scans revealed candidate genes linked to metabolism-Zinc Finger Ran-Binding Protein 3 (*ZRANB3*), growth-Sortilin Related VPS10 Domain Containing Receptor 1 (*SORCS1*), reproduction–Sus Scrofa Chromosome 9 quantitative trait loci (SSC9 QTLs), and immunity-Tudor Domain-Containing Protein 3 and CKLF-like MARVEL transmembrane Domain Containing 7 (*TDRD3*, *CMTM7*), reflecting adaptation to tropical production systems. Our results provide a comprehensive genomic characterisation of the ADP within a global context, revealing both genetic richness and vulnerability to genetic erosion. These findings underscore the importance of structured breeding and conservation strategies in preserving this unique African genetic resource and supporting sustainable pig production under changing climatic conditions.

## 1. Introduction

Pigs (*Sus scrofa domesticus*) rank among the world’s most widely raised livestock, accounting for ~36% of global meat production [1]. Their importance rests on key traits including rapid growth, short generation intervals, and high feed conversion efficiency, making them central pillars of intensive livestock production systems, particularly in Europe and Asia. Modern commercial breeds such as Large White, Landrace, and Duroc dominate global pork supply chains. In contrast, indigenous pig breeds persist largely within smallholder and subsistence-based systems, where they often exhibit unique adaptive traits including heat tolerance, resilience to endemic diseases, and capacity to thrive under low-input husbandry, which are critical in the face of rising food insecurity and climate change [2,3,4].

Pig domestication is a complex, multi-regional process. Archaeological and genetic evidence pinpoint the initial domestication event to Southwest Asia around 8000–10,000 years ago, followed by secondary and independent domestication in Europe and East Asia [5,6,7]. Early domestic pigs migrated with human populations and interbred with local wild boar, leading to region-specific admixture patterns [5,8]. European pig breeds themselves have been shaped by a mix of wild boar heritage and later introgression from Asian domestic pigs during selective breeding initiatives [9]. Although Eurasian pigs have been extensively studied, African pig populations remain less well understood [10,11]. Multiple hypotheses continue to explain their origins: introductions via Near Eastern agricultural expansions through Egypt [10], possible independent domestication events within Africa, and gene flow associated with European colonial livestock imports [12]. Genomic data so far have been inconclusive, underscoring the need for high-resolution SNP-based studies to clarify African pig ancestry [11,13].

The broader Suidae family in Africa includes wild relatives such as the warthog (*Phacochoerus africanus*), bushpig (*Potamochoerus porcus*), and giant forest hog (*Hylochoerus meinertzhageni*)—lineages crucial to understanding pig evolution and adaptation [13,14]. Within sub-Saharan smallholder systems, indigenous pigs offer invaluable resilience to environmental and disease pressures. Yet, these populations face increasing genetic dilution due to indiscriminate crossbreeding with exotics (imported breeds) such as Large White, Landrace, Hampshire, and Pietrain [1,15]. The Ashanti Dwarf pig (ADP) of Ghana exemplifies this trend. Historically valued for its hardiness and adaptability, the ADP has seen declines in both numbers and genetic integrity since the 1980s, driven by unsystematic crossbreeding with imported commercial breeds [16]. Morphologically, ADPs are distinct, typically small-bodied, black-coated, and suited to extensive low-input management. It exhibits moderate reproductive performance, including relatively small litter sizes but good mothering ability, and is valued for its disease tolerance and ability to thrive under extensive management conditions [16,17]. Yet, their genetic relationships to global pig populations remain poorly defined.

Advances in genomics, especially high-density SNP arrays (e.g., the Illumina PorcineSNP60 and SNP80 BeadChips), have transformed the study of pig genetic diversity, inbreeding, LD, and selection [18,19]. While Kenyan indigenous pigs have been characterised using SNP-based approaches [13], this kind of analysis has not yet been applied to West African breeds like the ADP. A recent study by [20] employed the PorcineSNP60K BeadChip to investigate Ghanaian pig populations, including the ADP, revealing moderate heterozygosity (0.28 in ADPs vs. 0.31 in exotics), regional clustering via PCA (north vs. south), evidence of admixture in ADMIXTURE analysis, and FST-linked SNPs associated with growth and disease resilience QTLs.

This study aims to build on and extend these findings by conducting a genome-wide analysis of the ADP against African, European, and Asian pig populations. Among others, we sought to assess levels of genetic diversity and inbreeding in ADP populations, characterise population structure and admixture relative to some global pig breeds, and identify genomic regions under selection that may underpin adaptive traits in the ADP. By situating the ADP within a broader genomic context, this work not only clarifies its genetic uniqueness but also informs conservation strategies and reinforces its potential as a genetic resource for sustainable pig production in sub-Saharan Africa.

## 2. Materials and Methods

### 2.1. Data Sources and Populations

To assess the genetic relationships of Ashanti Dwarf pigs (ADP) with other global pig populations, we conducted a genetic analysis combining SNP datasets from Africa, Europe, and Asia. The Ghanaian ADP dataset was merged with publicly available genotype data from Kenyan indigenous pigs (Busia and Homabay), wild suids (warthog, bushpig, and wild boar), and European commercial breeds (Yorkshire, Large White, Landrace, and Duroc) [13]. In addition, SNP data from four Chinese breeds (Jinhua, Jiangquhai, Meishan, and Xian) were retrieved from a larger dataset of over 2000 pigs. These breeds were selected based on the availability of publicly accessible high-density SNP datasets (PorcineSNP60K), ensuring compatibility with other populations genotyped using PorcineSNP60K and PorcineSNP80K platforms. The aim was to represent major East Asian domestic pig lineages within a global comparative framework [21]. Kenyan pigs were genotyped using the Illumina PorcineSNP60 and PorcineSNP80 BeadChips (Illumina Inc., San Diego, CA, USA) [13], Chinese pigs were genotyped using the PorcineSNP60K assay [22]. In contrast, European commercial breeds and wild suids were genotyped using either the SNP60 or SNP80 platforms. The final dataset comprised 875 individuals across 19 populations. Detailed information on breed composition and sample sizes per population is provided in Table S1. All breeds in the merged dataset were included in population structure and admixture analyses. For genetic diversity and inbreeding analyses, ADP, Bush pigs, Busia, Homabay, Jinhua, Warthog, Wild boar, Yorkshire, Large White, Landrace, Domestic pig (a mixed population of non-breed-specific domestic pigs obtained from published datasets), and Duroc were used as regional representative breeds to facilitate clearer comparisons across populations.

### 2.2. Quality Control and Dataset Merging

Quality control of genotype data was conducted in PLINK v1.9 [23]. SNPs with more than 5% missing genotype calls were excluded, and variants with a minor allele frequency (MAF) below 0.01 were removed to minimise the influence of rare alleles on downstream analyses. After filtering, a total of 59,124 high-quality SNPs were retained, yielding an overall genotyping rate of 99.3%. Because the datasets originated from multiple studies and genotyping platforms, harmonisation steps were implemented to ensure consistency across sources. SNPs were matched using dbSNP reference SNP identifiers (RSIDs) to resolve naming discrepancies, and multiallelic markers as well as duplicate SNPs were removed. Strand inconsistencies were corrected using PLINK strand-flip procedures, following established best practices for integrating cross-study genomic datasets [24]. These steps ensured that all datasets were aligned to a common reference orientation and suitable for combined analyses.

### 2.3. Genetic Diversity Within Populations Analysis

To evaluate genomic diversity within each population, we calculated a suite of complementary diversity metrics. These included the proportion of polymorphic SNPs, distributions of minor allele frequency (MAF), observed heterozygosity (HO), expected heterozygosity (HE), and Wright’s inbreeding coefficient (FIS). Collectively, these parameters provide a robust assessment of allelic richness, genetic variability, and departures from the Hardy–Weinberg equilibrium within populations. All diversity statistics were computed in PLINK v1.9 [23] using the standard genotype-based algorithms implemented in the software.

### 2.4. Linkage Disequilibrium (LD) and LD Decay

Pairwise linkage disequilibrium (LD) was quantified for all populations by estimating the squared correlation coefficient (r^2^) between SNP pairs using PLINK v1.9, implementing a sliding-window approach with a 1 Mb window size. This procedure captures local patterns of allelic association while accounting for variation in marker density across the genome. To characterise LD decay, mean r^2^ values were calculated across successive physical distance classes and plotted against increasing inter-marker distances. Distance bins ranged from 50 kb to 1000 kb, allowing visualisation of both short-range and long-range LD patterns. This analytical framework follows established procedures commonly applied in pig genomic studies [18,25] and enables comparison of historical recombination rates and demographic processes among populations.

### 2.5. Runs of Homozygosity (ROH) and Genomic Inbreeding

Runs of homozygosity (ROH) were detected using the sliding-window approach implemented in PLINK v1.9. To ensure reliable identification of autozygous segments, we applied stringent filtering criteria, requiring a minimum of 50 consecutive SNPs per ROH, a maximum of five missing genotypes per window, and a marker density exceeding one SNP per 100 kb. Only segments with a minimum physical length of 1 Mb were retained to exclude short tracts that may arise from linkage disequilibrium rather than true identity-by-descent. For each individual, both the total number of ROH and the mean ROH length were calculated to characterise recent versus ancient inbreeding signatures. Genomic inbreeding coefficients (FROH) were subsequently estimated as the proportion of the autosomal genome encompassed by ROH, following established approaches in livestock genomics [26,27]. This metric provides a robust measure of autozygosity and allows comparison of inbreeding levels across populations with differing demographic histories.

### 2.6. Population Structure Analysis

Population structure was assessed using complementary multivariate and model-based clustering approaches. Principal component analysis (PCA) was performed in R v3.5 [28] using the ‘prcomp’ function on pruned SNP genotype data, allowing for the visualisation of the major axes of genetic differentiation among individuals and populations. PCA provided an unbiased representation of broad-scale structure and enabled the identification of clusters consistent with geographical origins and known breed histories. To further quantify individual ancestry proportions, we applied the model-based algorithm implemented in ADMIXTURE v1.3.0 [29]. Analyses were conducted for a range of hypothesised ancestral populations (K), and five-fold cross-validation was used to identify the most likely number of clusters by selecting the K value with the lowest cross-validation error. The resulting admixture coefficients provided high-resolution insights into shared ancestry, introgression patterns, and population relationships across African, European, and Asian pig groups.

### 2.7. Genetic Differentiation and Detection of Selection Signatures

Genetic differentiation among populations was quantified using Wright’s fixation index (FST) [30], providing a measure of allele frequency divergence attributable to population structure. To identify genomic regions potentially subject to divergent or recent selection, we applied two complementary selection scan approaches that capture both inter-population and intra-population signals.

First, FST outlier analysis was conducted by calculating per-SNP FST values across populations. SNPs falling within the top 1% of the empirical FST distribution were considered putative candidates for divergent selection, following established thresholds used in livestock genomics [31]. This method highlights loci exhibiting unusually high differentiation relative to the genomic background.

Second, within-population selection was evaluated using the integrated haplotype score (iHS) approach, which detects signatures of incomplete selective sweeps based on extended haplotype homozygosity. iHS calculations were performed using the REHH v3.1.2 package in R [32,33]. SNPs with |iHS| values within the top 1% of the genome-wide distribution were retained as strong candidates for recent positive selection.

For both approaches, candidate regions were further examined by annotating genes overlapping or proximal to significant SNPs using Ensembl BioMart for the Sus scrofa 11.1 reference genome [34].

## 3. Results

### 3.1. Quality Control

A total of 61,565 SNPs were initially available in the Ghanaian dataset, of which 59,302 markers passed quality-control thresholds for call rate, minor allele frequency, and Hardy–Weinberg equilibrium. Following dataset harmonisation and merging with genotypes from Kenyan, European, and Asian pig populations, a final panel of 875 individuals genotyped at 59,124 shared SNPs was obtained. The merged dataset exhibited a high overall genotyping rate of 99.27%, providing a robust foundation for downstream population genomic analyses.

### 3.2. Genetic Diversity Within Populations

Genetic diversity metrics for all populations are presented in Table 1. The Ashanti Dwarf Pig (ADP) and the Kenyan Busia population exhibited the highest levels of genomic variability, with more than 98% of SNPs being polymorphic and mean minor allele frequencies (MAF) ranging from 0.26 to 0.27. These values indicate substantial allelic richness and are characteristic of populations with large historical effective sizes. In contrast, wild suids, including Warthog and Bush Pig, showed markedly reduced diversity, with polymorphic SNP proportions of 81–83% and lower MAF values (0.013–0.15). This reduced variability likely reflects their greater evolutionary divergence from domestic pigs and smaller population sizes.

Observed heterozygosity (HO) was highest in Busia pigs (0.33), followed by the ADP (0.28), consistent with their high levels of polymorphism. Expected heterozygosity (HE) showed similar trends across populations. Notably, the ADP displayed the highest inbreeding coefficient (FIS = 0.15), approximately double that observed in the Kenyan pig populations, indicating a greater degree of non-random mating or recent demographic contraction. European (Yorkshire, Large White, Landrace and Duroc) and Chinese (Jinhua) breeds exhibited FIS values close to zero or slightly negative, patterns typically associated with managed breeding schemes designed to minimise inbreeding and maintain heterozygosity.

### 3.3. Linkage Disequilibrium and LD Decay

**Figure 1:**
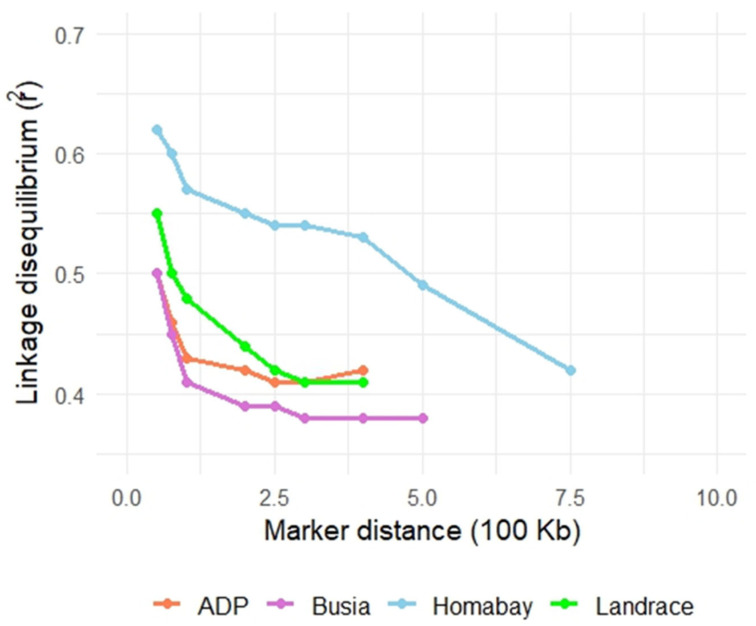
Linkage disequilibrium (LD) decay in ADP, Busia, Homabay, and Landrace populations.

Linkage disequilibrium (LD) decay patterns for the four representative populations are presented in Figure 1. The Ashanti Dwarf Pig (ADP) and the Kenyan Busia population exhibited broadly similar LD decay trajectories, with moderate initial LD levels and relatively rapid declines over short inter-marker distances. In contrast, the Homabay population showed markedly higher LD values across all distances, closely resembling the profile of the commercial Landrace breed. This pattern suggests reduced historical recombination and a more restricted breeding structure in Homabay pigs, consistent with a more “closed” population system. Across all populations, mean r^2^ values exceeded 0.50 at marker distances ≤50 kb, reflecting short-range LD typical of pig genomes. LD declined progressively with increasing physical distance, although the rate of decay differed among populations, with Landrace and Homabay showing shallower decay slopes than ADP and Busia. Chromosome-level LD estimates revealed notable heterogeneity, with the highest LD observed on chromosome 14 and the lowest on chromosome 10 (Table 2), consistent with previous findings linking chromosome 14 to selection on production-related quantitative trait loci (QTL).

### 3.4. Runs of Homozygosity

Runs of homozygosity (ROH) varied substantially across populations, reflecting differences in demographic history, breeding structure, and recent inbreeding (Table 3). The Ashanti Dwarf Pig (ADP) exhibited the highest average number of ROH per individual (46.7 ± 18.7), as well as the longest cumulative ROH length (mean = 352.6 Mb). These values correspond to a genomic inbreeding coefficient of FROH = 0.40, indicating extensive autozygosity and suggesting recent inbreeding events, likely driven by small effective population sizes and non-random mating within low-input production systems. Busia pigs displayed similarly elevated levels of genomic inbreeding (FROH = 0.398), with an average of 34.1 ± 13.1 ROH segments per individual and mean ROH lengths of 249.6 Mb. In contrast, the Homabay population showed exceptionally low ROH levels (mean ROH count: 0.97 ± 0.92; mean length: 3.4 Mb), corresponding to FROH = 0.003. This pattern reflects minimal recent inbreeding and suggests a more open breeding structure or greater historical gene flow relative to the other African pig populations—ADP and Busia pigs. Among the commercial breeds, ROH patterns reflected known breeding histories. Landrace, Large White, and Yorkshire pigs exhibited moderate ROH counts and lengths, consistent with structured but genetically managed breeding programmes aimed at balancing selection and heterozygosity. Domestic pigs (mixed population) displayed intermediate ROH levels (FROH = 0.092). Chinese Jinhua pigs showed relatively low ROH burdens, reflective of their distinct demographic history and reduced selection intensity. Wild suids displayed the most variable ROH profiles. Warthogs exhibited extremely high ROH variance (0–213 segments; ROH length 0–1320.9 Mb) and the highest genomic inbreeding estimate (FROH = 0.574), likely reflecting small, isolated populations with long-term drift. Bush pigs and wild boar showed comparatively low ROH burdens and low inbreeding coefficients. Overall, these results highlight the contrasting demographic pressures acting on indigenous African pigs, with ADP and Busia showing evidence of substantial recent inbreeding, while Homabay pigs remain largely outbred.

### 3.5. Population Structure

**Figure 2:**
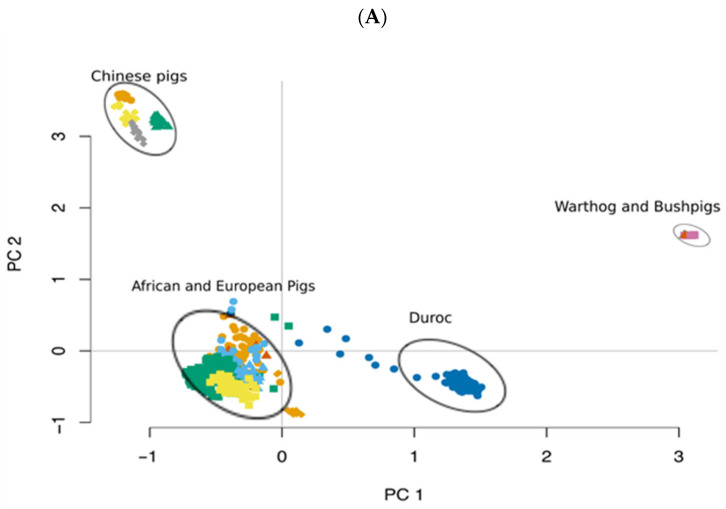

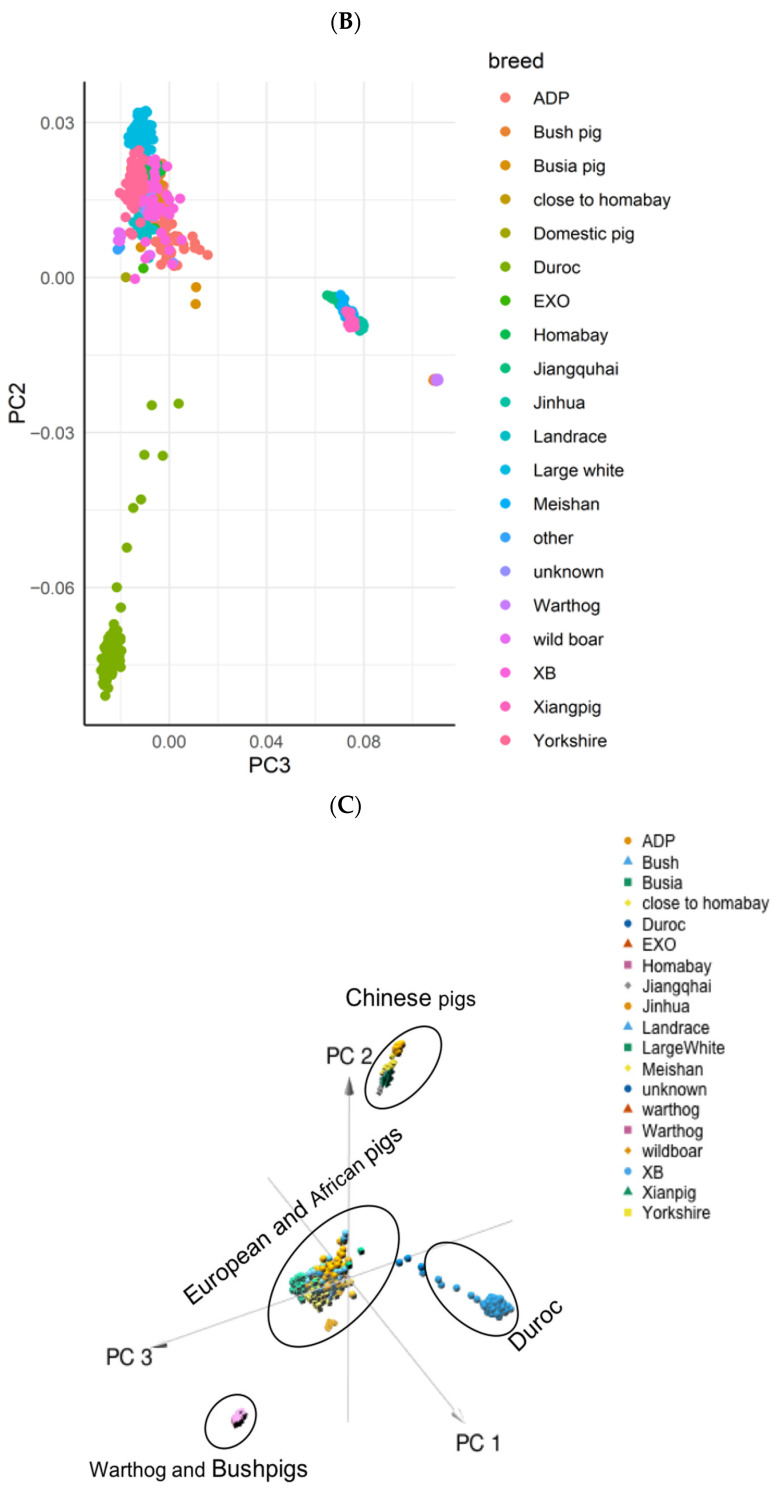
(**A**–**C**). Principal component analysis of the selected global pig populations. (**A**) PC1 vs. PC2 separates African, European, and Asian populations. (**B**) PC1 vs. PC3 highlights the separation of wild suids. (**C**) 3D PC plot of PC1 vs. PC2 vs. PC3 showing additional separation among populations. Each point represents an individual (n = 875) shown in parentheses.

**Figure 3:**
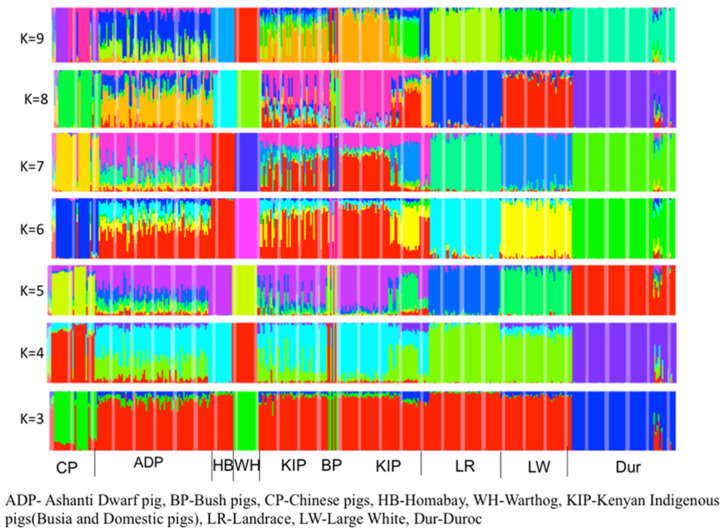
ADMIXTURE analysis (K = 1–10). Each bar represents an individual (n = 875), partitioned into ancestry proportions. Colours correspond to inferred ancestral clusters. Cross-validation error indicated optimal clustering at K = 6.

Principal component analysis (PCA) revealed a clear population structure largely driven by geographic origin (Figure 2). ADPs clustered closely with Busia and Homabay pigs and were positioned nearer to European breeds than to Chinese populations. In contrast, warthog and bush pig samples formed discrete, well-separated clusters, reflecting their deep divergence from domestic lineages. ADMIXTURE analysis supported the patterns observed in the PCA (Figure 3). At K = 6, both ADP and Busia populations exhibited notable admixture components derived from European and Chinese ancestries. In contrast, the Homabay pigs displayed a largely homogeneous genetic profile with minimal evidence of introgression.

### 3.6. Genetic Differentiation and Signatures of Selection

**Figure 4:**
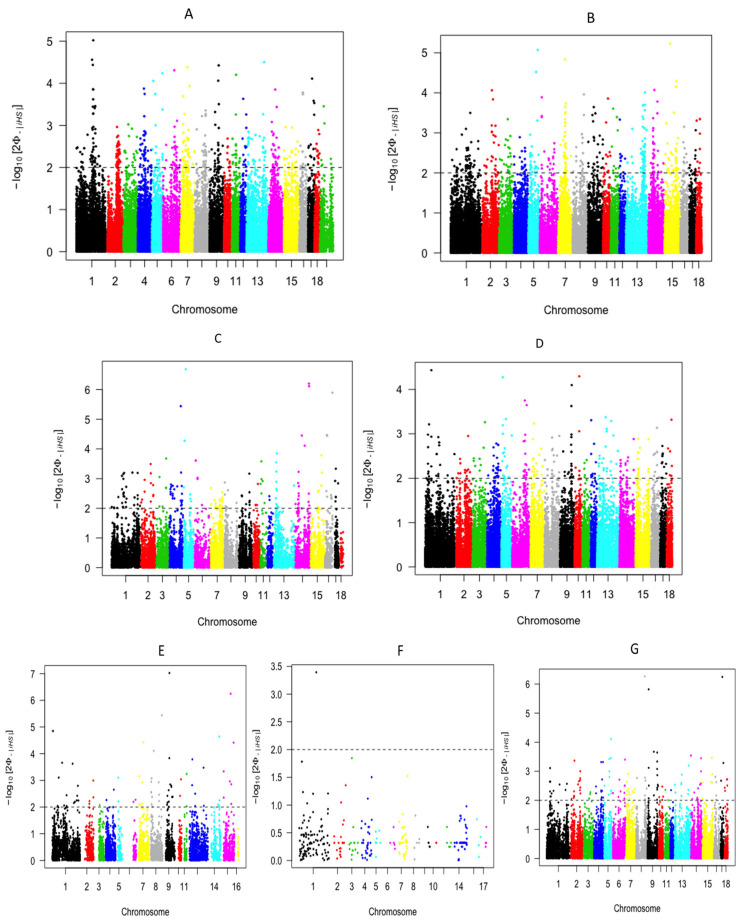
Selection signals on the autosomal chromosomes 1–18 of the pig populations studied ((**A**) Ashanti Dwarf pigs, (**B**) Busia, (**C**) Homabay, (**D**) Landrace, (**E**) Bush pig, (**F**) Warthog, (**G**) Jinhua).

Pairwise FST analyses highlighted moderate to high differentiation among the populations (mean FST = 0.20). ADPs were most similar to Busia pigs (FST = 0.017) and most differentiated from Jinhua pigs (FST = 0.89), consistent with the clustering and admixture results (see Tables S2 and S3). Genome-wide FST scans revealed multiple outlier regions in ADPs (Figure 4). Candidate genes included SORCS1 (growth and metabolism), ZRANB3 (feed efficiency), LRP1B (backfat deposition), and CA8 (neurological traits). Importantly, many of these loci overlapped with quantitative trait loci (QTLs) for economically significant traits, suggesting strong selection pressures (Figure 4). Integrated haplotype score (iHS) analyses provided additional insights into recent selection events. In ADPs, iHS outliers were enriched for genes related to immunity (TDRD3, CMTM7), reproduction, and metabolism (see Table S4). Busia pigs displayed similar patterns, with candidate regions overlapping those in ADPs, particularly in immune function genes (Table S4). Homabay pigs also showed immune-related candidates but fewer overlaps, suggesting a more distinct evolutionary trajectory (see Table S4). By contrast, Jinhua (Chinese) pigs displayed iHS outliers primarily associated with production-related pathways such as growth rate and fat deposition (Table S5). In contrast, Landrace (European) pigs showed signatures in regions linked to domestication traits and intensive breeding (see Table S6).

Overlap analyses demonstrated that ADPs and Busia pigs shared the most candidate loci, reflecting their close relationship and possible shared adaptive pressures in smallholder systems. Homabay pigs shared fewer regions with ADPs and Busia, highlighting population-specific adaptations (Table S4). Notably, shared African signals frequently mapped to immune and stress-response pathways, underscoring the importance of resilience traits in indigenous pigs.

## 4. Discussion

This study provides a comprehensive genomic characterisation of the Ashanti Dwarf Pig (ADP), placing this indigenous Ghanaian breed within the broader context of African, European, and Asian pig populations. By combining genome-wide SNP data with analyses of genetic diversity, linkage disequilibrium (LD), runs of homozygosity (ROH), and selection signatures, we generate new insights into the demographic history, adaptive potential, and conservation status of ADPs. Importantly, our findings underscore both the genetic richness and the vulnerability of this breed, with implications for breeding, adaptation, and food security in sub-Saharan Africa.

The ADP displayed high levels of polymorphism (~99%) and moderate expected heterozygosity, values comparable to other indigenous African pigs such as Busia (Kenya) but higher than observed in commercial Chinese breeds such as Jinhua. This is consistent with earlier reports suggesting that African pigs retain significant genetic variation despite centuries of local adaptation [13,20]. However, the relatively high inbreeding coefficients in ADPs (FIS = 0.15; FROH = 0.40) indicate ongoing risks of genetic erosion. Such levels of homozygosity are typically associated with small effective population sizes and mating among relatives; patterns commonly observed in low-input production systems where pigs are raised under free-range or semi-scavenging conditions [17]. Comparable trends have been documented in other African contexts. For example, South African indigenous pigs show higher genomic diversity than commercial breeds but also carry signals of local inbreeding [35]. A recent review by [36] highlights that while local African pigs are resilient and well-adapted to climate change, unmanaged crossbreeding threatens their genetic integrity. Together, these studies suggest that without formal breeding schemes, valuable alleles unique to indigenous populations, such as the ADP, could be lost.

Patterns of LD decay and ROH provide windows into the demographic history of populations. ADPs and Busia pigs showed similar LD decay trajectories, suggesting parallel histories of genetic drift and recombination. In contrast, Homabay pigs displayed higher LD at shorter marker distances, resembling commercial Landrace pigs, which are known to have undergone more intensive selection. This indicates that Homabay pigs may represent a more “closed” genetic system compared with the more admixed and diverse ADPs. Chromosome 14 consistently exhibited elevated LD across populations, a pattern previously associated with selection on quantitative trait loci (QTL) for fat composition and meat quality [37]. Such signatures may reflect ongoing selection pressures in both indigenous and commercial contexts.

The ROH analysis further revealed long homozygosity tracts in ADPs, consistent with recent inbreeding. This pattern echoes observations in other livestock species under limited management. For example, in South African pigs, long ROH blocks have been linked to both genetic isolation and recent artificial selection [35]. Similarly, Ref. [38] demonstrated that ROH contributes significantly to domestication processes and fitness outcomes in pigs, underscoring their value as markers of demographic history. The consistency between ROH-based inbreeding and Wright’s FIS estimates in our study reinforces the robustness of these findings. Our findings contrast with the low genomic inbreeding (FROH > 2 Mb = 0.043) reported by [39] in Ugandan smallholder pigs. Their low inbreeding levels were attributed to frequent boar exchange and stock turnover following disease outbreaks, which prevent mating among relatives. Although referred to as “local” (LOC) pigs, their report suggests Ugandan pigs are likely derived from old British and modern European breeds with introgressed *MS* haplotypes [10,40].

Population structure analyses revealed clear geographic clustering. ADPs grouped with other African pigs but remained closer to European commercial breeds than to Chinese pigs. This supports earlier work using mitochondrial and SNP data, which suggested both European and Asian ancestry in Ghanaian pigs [17,20]. At higher K values in the ADMIXTURE analysis, evidence of introgression from European and, to a lesser extent, Chinese pigs became apparent, reflecting recent gene flow from the introduction of exotic breeds for productivity gains.

The role of colonial and post-colonial livestock introductions in shaping African pig genomes has been widely debated. Ref. [41] hypothesised that European breeds were brought into Africa during the colonial era, while others suggest earlier introductions via the Nile corridor [10]. Our findings of admixture in ADPs are consistent with these scenarios, though the persistence of unique African genetic signatures demonstrates the resilience of indigenous pig populations despite historical gene flow. Interestingly, the Homabay pig population remained genetically distinct, showing minimal admixture even at high K values. This suggests either geographic isolation or stronger cultural preferences for maintaining local lineages. Such variation within African pigs emphasises that conservation strategies should be region-specific and account for local histories of breed management.

Selection scans identified candidate genes associated with growth, reproduction, metabolism, and immunity. For instance, *SORCS1* and *ZRANB3* were highlighted in ADPs. *SORCS1* has been linked to growth and metabolism [42], while *ZRANB3* has been implicated in feed efficiency in cattle [43]. These associations suggest that natural and farmer-driven selection in ADPs may have targeted traits critical for productivity under resource-limited systems. Immune-related genes such as *TDRD3* and *CMTM7* were also under strong selection. *TDRD3* has been associated with chromatin regulation and immunity in humans [44], while *CMTM7* is implicated in B-cell survival and immune function [45]. The detection of these genes in ADPs supports anecdotal reports of their resilience against endemic diseases, a trait that is invaluable for smallholder farmers in tropical environments.

Our findings align with broader studies of selection in African pigs. Ref. [35] identified signatures of selection in South African pigs for both production and disease resistance traits. Similarly, ref. [46] reported strong adaptive signals in heat-stress pathways such as *AMPK* and *mTOR* in pigs from harsh environments. Genome-wide scans in Chinese and other indigenous pigs have also highlighted convergent selection on traits related to reproduction, immunity, and environmental resilience [47,48]. The overlap of selection signals between ADPs and other African populations strengthens the case that these alleles represent genuine adaptive features rather than stochastic drift. Importantly, the *CA8* locus, identified in ADPs, has previously been linked to behavioural and neurological traits in mammals [49], raising intriguing questions about its role in local adaptation.

Taken together, these findings have significant implications for both conservation and applied breeding. The ADP retains substantial genetic diversity, but high inbreeding levels present risks for long-term viability. Structured breeding schemes that minimise inbreeding while maintaining genetic variability are urgently needed. Without intervention, the continued introduction of exotic breeds could erode unique adaptive alleles, which is a concern echoed by [36] in their review of African pig conservation. At the same time, the identification of adaptive loci highlights the potential of ADPs as reservoirs of beneficial alleles. These could be strategically introgressed into global pig breeding programmes to improve traits such as disease resistance, heat tolerance, and feed efficiency. Given the challenges posed by climate change, such alleles may become increasingly valuable in commercial systems worldwide.

Finally, the ADP exemplifies the dual challenges and opportunities inherent in managing indigenous livestock. On one hand, it is a repository of unique genetic diversity shaped by centuries of adaptation to local environments. On the other hand, it faces threats from inbreeding, admixture, and changing production systems. By situating ADPs within global pig diversity, our study highlights the urgent need for integrative strategies that balance conservation with sustainable utilisation. Ultimately, genomic insights such as those presented here provide the evidence base needed to design breeding programmes that preserve genetic diversity while enhancing productivity. In doing so, they ensure that indigenous breeds like the ADP continue to play a vital role in food security, cultural identity, and resilience in Africa and beyond.

## 5. Conclusions

This study provides a comprehensive genomic analysis of the Ashanti Dwarf pig (ADP) relative to other African, European, and Asian pig populations. Using high-density SNP data, we demonstrate that ADPs retain substantial genetic diversity yet face elevated levels of inbreeding and admixture with exotic breeds. Patterns of linkage disequilibrium and runs of homozygosity highlight both recent demographic pressures and the persistence of unique African genomic signatures. Population structure analyses reveal that ADPs cluster with African and European breeds but also show evidence of introgression from Chinese pigs, reflecting complex historical and recent gene flow. Importantly, signatures of selection identified candidate genes associated with metabolism, reproduction, immunity, and adaptation to tropical environments. These findings underscore the potential of ADPs as reservoirs of valuable alleles for resilience, productivity, and disease resistance in low-input systems. The results highlight an urgent need for structured breeding and conservation strategies to preserve the genetic integrity of ADPs. Protecting this indigenous genetic resource will not only safeguard cultural heritage and biodiversity in Ghana but also contribute to global efforts to enhance livestock resilience under climate change. In summary, the ADP exemplifies both the challenges and opportunities of indigenous livestock management: while at risk from inbreeding and admixture, it remains a vital source of adaptive genetic variation. Harnessing this potential through conservation-oriented breeding programmes will be key to ensuring sustainable pig production in Africa and beyond. These findings underscore the urgency for timely conservation action.

### Limitations

While this study combines multiple publicly available SNP genotype datasets, variations in genotyping platforms, SNP density, and sample sizes across studies may have influenced marker comparability and downstream analyses. In particular, some populations had limited representation (e.g., Homabay, *n* = 14), which may reduce the statistical power of selection scans and population structure inference. Future work that incorporates whole-genome sequence data, uniform genotyping methods, and larger sample sizes will enable higher-resolution insights into demographic history, adaptive variation, and functional diversity in African pig populations.

## Figures and Tables

**Table 1:** Genetic diversity parameters across pig populations.

Populations	*N*	%SNPS > 0.05	MAF ± SD	H_O_ ± SD	H_E_ ± SD	*F*_IS_ ± SD
ADP	106	0.99	0.26 ± 0.15	0.28 ± 0.14	0.34 ± 0.16	0.15 ± 0.17
Bush pigs	14	0.83	0.15 ± 0.14	0.10 ± 0.15	0.19 ± 0.17	0.60 ± 0.57
Busia pigs	174	0.98	0.27 ± 0.14	0.33 ± 0.14	0.36 ± 0.14	0.07 ± 0.12
Homabay	14	0.99	0.18 ± 0.16	0.22 ± 0.19	0.25 ± 0.19	0.09 ± 0.23
Warthog	32	0.81	0.013 ± 0.07	0.02 ± 0.10	0.02 ± 0.08	0.45 ± 0.18
Wild boar	11	0.99	0.18 ± 0.17	0.18 ± 0.18	0.24 ± 0.20	0.24 ± 0.09
Yorkshire	100	0.99	0.26 ± 0.15	0.35 ± 0.16	0.34 ± 0.15	−0.02 ± 0.06
Large White	100	0.99	0.24 ± 0.15	0.33 ± 0.17	0.32 ± 0.16	−0.03 ± 0.04
Landrace	25	0.98	0.25 ± 0.15	0.32 ± 0.18	0.32 ± 0.16	0.00 ± 0.04
Domestic pig	13	0.96	0.27 ± 0.14	0.37 ± 0.19	0.36 ± 0.15	−0.05 ± 0.17
Duroc	144	0.96	0.20 ± 0.16	0.28 ± 0.19	0.28 ± 0.18	−0.05 ± 0.31
Jinhua	17	0.99	0.10 ± 0.15	0.14 ± 0.2	0.13 ± 0.19	−0.05 ± 0.12

**Table 2:** Average r^2^ values across autosomes for selected populations.

Chromosome	Average r^2^ ADP	Average r^2^ Busia	Average r^2^ Homabay	Average r^2^ Landrace
1	0.48 ± 0.23	0.52 ± 0.25	0.68 ± 0.28	0.60 ± 027
2	0.45 ± 0.22	0.49 ± 0.23	0.70 ± 0.29	0.56 ± 0.28
3	0.44 ± 0.22	0.46 ± 0.22	0.64 ± 0.28	0.60 ± 027
4	0.47 ± 0.23	0.49 ± 0.24	0.72 ± 0.28	0.56 ± 0.28
5	0.47 ± 0.23	0.50 ± 0.25	0.70 ± 0.28	0.56 ± 0.28
6	0.45 ± 0.22	0.49 ± 0.25	0.67 ± 0.29	0.56 ± 0.28
7	0.44 ± 0.21	0.49 ± 0.24	0.63 ± 0.29	0.56 ± 0.28
8	0.46 ± 0.22	0.47 ± 0.22	0.70 ± 0.30	0.56 ± 0.28
9	0.46 ± 0.22	0.47 ± 0.23	0.65 ± 0.30	0.56 ± 0.28
10	0.43 ± 0.21	0.45 ± 0.21	0.59 ± 0.28	0.56 ± 0.28
11	0.46 ± 0.23	0.48 ± 0.25	0.72 ± 0.27	0.56 ± 0.28
12	0.45 ± 0.21	0.45 ± 0.22	0.75 ± 0.28	0.56 ± 0.28
13	0.48 ± 0.23	0.51 ± 0.25	0.71 ± 0.29	0.56 ± 0.27
14	0.50 ± 0.24	0.52 ± 0.25	0.76 ± 0.27	0.56 ± 0.28
15	0.45 ± 0.22	0.48 ± 0.24	0.75 ± 0.29	0.56 ± 0.28
16	0.43 ± 0.20	0.45 ± 0.22	0.68 ± 0.29	0.56 ± 0.28
17	0.44 ± 0.21	0.46 ± 0.22	0.67 ± 0.29	0.56 ± 0.28
18	0.44 ± 0.23	0.47 ± 0.23	0.71 ± 0.28	0.56 ± 0.28
Overall	0.46 ± 0.23	0.49 ± 0.24	0.69 ± 0.29	0.56 ± 0.28

**Table 3:** Runs of homozygosity (ROH)-based metrics across populations.

Population	Number of ROH per Individual			Length of ROH (Mb) per Individual			Inbreeding Based on ROH
	Mean	Min	Max	Mean	Min	Max	FROH
ADP	46.7 ± 18.7	16	113	352.6	540.1	931.1	0.40
Busia	34.1 ±13.1	0	74	249.6 ± 193.3	0	916.9	0.398
Homabay	0.97 ± 0.92	0	2	3.4 ± 3.6	0	7.6	0.003
Domestic pig	13.5 ± 9.2	1	34	72.4 ± 64.1	1.8	211.6	0.092
Landrace	19.9 ± 4.5	8	27	109.3 ± 41.6	42.7	193.8	0.083
Large white	20.4 ± 6.3	9	40	108.9 ± 61.1	30.3	327.7	0.142
Warthog	13.2 ± 51.9	0	213	78.8 ± 310.4	0	1320.9	0.574
Wild boar	21.8 ± 8.2	8	40	8.1 ± 9.6	1.9	92.9	0.04
Yorkshire	19.2 ± 5.7	1	36	5.3 ± 5.8	1.2	70.5	0.03
Duroc	25.3 ± 10.2	0	50	5.9 ± 4.7	1.2	53.5	0.02
Jinhua	8.2 ± 3.1	3	15	10.0 ± 8.5	2.7	63.2	0.03
Bush pigs	45.6 ± 62.0	0	145	8.06 ± 5.8	1.78	46.4	0.02

## Data Availability

This manuscript includes data derived from the doctoral thesis of Sethlina Aryee, titled “Genome Analysis of the Ashanti Dwarf Pig of Ghana” (University of Cambridge). The thesis is publicly available, and there are no copyright issues associated with the reuse of this material. The datasets generated and/or analysed during the current study are available in the figshare repository, 10.6084/m9.figshare.30565946.

## Supplementary Materials

The following supporting information can be downloaded at: https://www.mdpi.com/article/10.3390/life16050745/s1; Table S1: Population composition, breed description, and SNP platforms used in this study; Table S2: Top FST values of ADP vs. Kenya vs. Chinese vs. Europe; Table S3: Top FST values for ADP vrs Kenyan Busia and Homabay pigs; Table S4: Combined ADP, Busia and Homabay top 1% within loci; Table S5:Top 1% iHS values > 2.5 in the Jinhua population representing the chinese populations; Table S6: Top 1% of iHS values in the Landrace population representing European breed.

## Abbreviations

ADPs	Ashanti Dwarf pigs
FST	Fixation index (variance of allele frequency among the total population)
FIS	Fixation index (measure of inbreeding)
FROH	Inbreeding coefficient based on Runs of Homozygosity
iHS	Integrated Haplotype Score
LD	Linkage Disequilibrium
PCA	Principal Component Analysis
QTL	Quantitative Trait Loci
SNPs	Single Nucleotide Polymorphisms
ROH	Runs of Homozygosity
